# Metagenome sequencing and recovery of 444 metagenome-assembled genomes from the biofloc aquaculture system

**DOI:** 10.1038/s41597-023-02622-0

**Published:** 2023-10-17

**Authors:** Meora Rajeev, Ilsuk Jung, Yeonjung Lim, Suhyun Kim, Ilnam Kang, Jang-Cheon Cho

**Affiliations:** 1 Department of Biological Sciences and Bioengineering, Inha University, Inharo 100, Incheon 22212, Republic of Korea; 2 Institute for Specialized Teaching and Research, Inha University, Inharo 100, Incheon 22212, Republic of Korea; 3 Center for Molecular and Cell Biology, Inha University, Inharo 100, Incheon 22212, Republic of Korea

**Keywords:** Metagenomics, Microbial ecology

## Abstract

Biofloc technology is increasingly recognised as a sustainable aquaculture method. In this technique, bioflocs are generated as microbial aggregates that play pivotal roles in assimilating toxic nitrogenous substances, thereby ensuring high water quality. Despite the crucial roles of the floc-associated bacterial (FAB) community in pathogen control and animal health, earlier microbiota studies have primarily relied on the metataxonomic approaches. Here, we employed shotgun sequencing on eight biofloc metagenomes from a commercial aquaculture system. This resulted in the generation of 106.6 Gbp, and the reconstruction of 444 metagenome-assembled genomes (MAGs). Among the recovered MAGs, 230 were high-quality (≥90% completeness, ≤5% contamination), and 214 were medium-quality (≥50% completeness, ≤10% contamination). Phylogenetic analysis unveiled *Rhodobacteraceae* as dominant members of the FAB community. The reported metagenomes and MAGs are crucial for elucidating the roles of diverse microorganisms and their functional genes in key processes such as nitrification, denitrification, and remineralization. This study will contribute to scientific understanding of phylogenetic diversity and metabolic capabilities of microbial taxa in aquaculture environments.

## Background & Summary

Uncultured microorganisms constitute a significant proportion of microbial populations in an ecosystem and play a vital role in its functioning^1^. The challenges associated with cultivating these microbes have constrained access to the vast phylogenetic and functional diversity they possess. However, recent advancements in metagenomics have opened a new window to explore the enigmatic “microbial dark matter”, revealing the hidden genetic potential of as-yet-uncultured microorganisms^2^.

One of the recent advancements in shotgun metagenomic data analysis is the generation of metagenome-assembled genomes (MAGs) through *de novo* assembly and binning of individual bacterial genomes from complex microbial communities^3^. This approach provides a culture-independent way to directly reconstruct genomes from environmental samples, thereby offering insights into the genomic makeup and metabolic potential of previously uncharacterized microbial taxa^4^. Since the first successful recovery of MAGs^5,6^, the approach has seen a remarkable expansion, with construction of hundreds to thousands of MAGs from a variety of complex environments, including thermal pools^7^, animal and human guts^8^, river estuaries^9^, deep marine sediments^10^, and activated sludge^11,12^. In fact, these MAGs have been used to explore the functional potential of microbes across various environments^12,13^.

Aquaculture is one of the fastest developing food sectors, meeting the global seafood demand^14^. As traditional open-water aquaculture systems encounter several challenges such as water pollution, disease outbreaks, and inefficient resource utilization, there is a growing need for sustainable and environmentally friendly aquaculture methods. In this context, biofloc technology (BFT) has emerged as a promising approach that facilitates recycling of toxic nitrogenous components into microbial biomass by supporting the growth of definite microbial consortia^15^.

The BFT-based aquaculture system principally relies on balancing the carbon-to-nitrogen (C/N) ratio to stimulate the growth of dense microbial aggregates (biofloc)^16^. The floc-associated bacterial (FAB) community helps regulate excessive nutrients, particularly inorganic nitrogen (e.g., ammonia and nitrite), by promoting heterotrophic assimilation. As organic matters accumulate in the biofloc aquaculture system, heterotrophic bacteria use these organic carbon compounds as a source of energy and simultaneously assimilate ammonia and nitrite into cellular components, including proteins and nucleic acids. Through this process, heterotrophic bacteria assimilate deleterious nitrogenous compounds into microbial biomass. This assimilated biomass subsequently serves as a valuable nutrient source for the culturing animals^17,18^.

In this manner, BFT systems not only maintains adequate water quality but also offers several other advantages, including enhanced productivity, regulation of animal health, and assurance of biosafety^19^. Since microbial communities determine the overall functioning of a BFT aquaculture system, substantial scientific efforts have been devoted to understanding the bacterial community composition of various BFT components^20–22^. However, most of these studies have used 16S rRNA gene amplicon sequencing (a metataxonomic approach), which provides information on community composition but falls short of capturing the complete genetic diversity and functional potential of microorganisms^23,24^. Therefore, earlier studies have recommended the employment of a metagenomic approach to investigate aquaculture systems^25^.

**Fig. 1:**
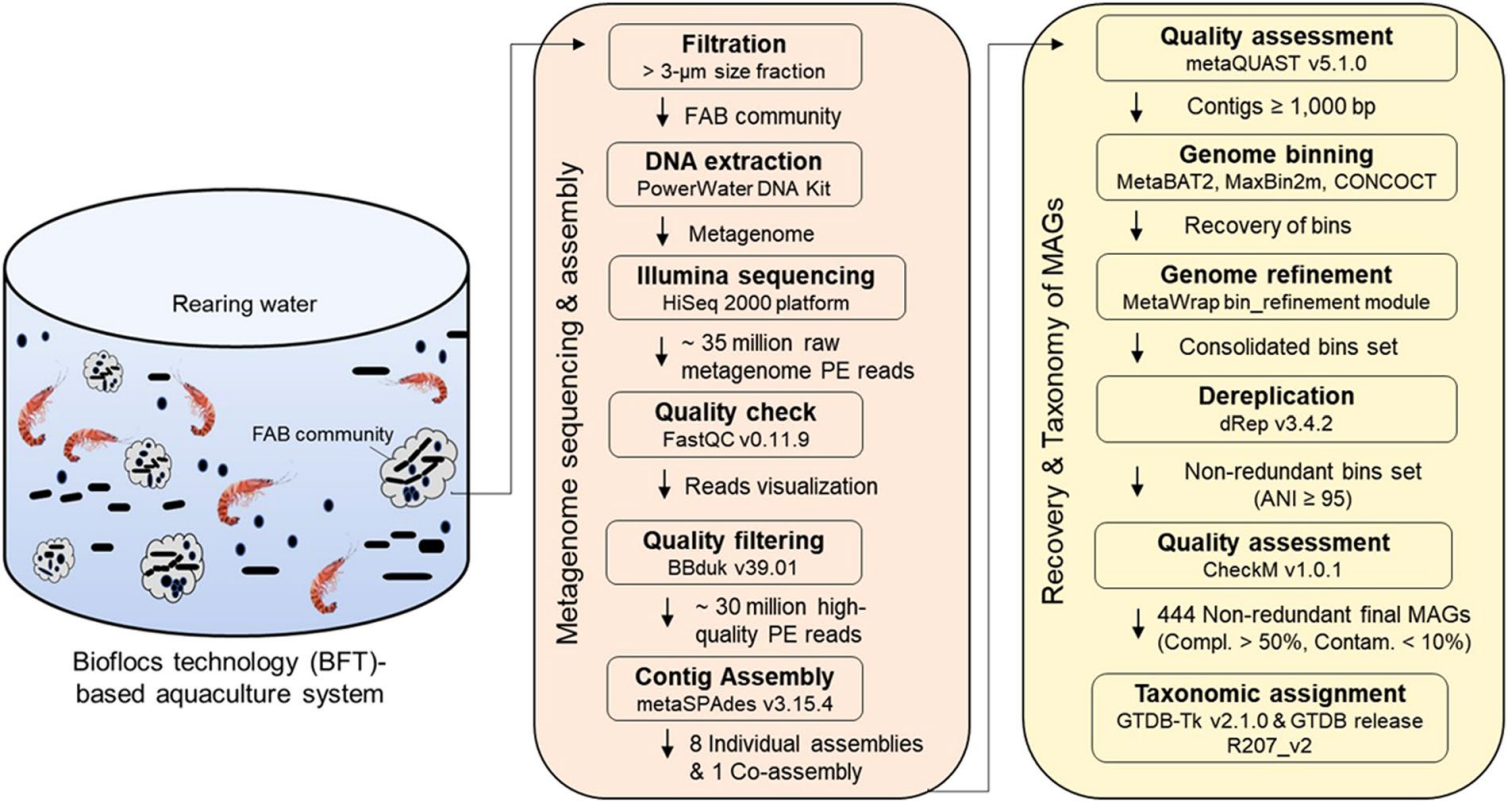
A schematic representation of methodological workflow. Figure illustrates major procedural steps followed for metagenome sequencing, assembly, and recovery of MAGs from the FAB community of a biofloc aquaculture system. Major methodological steps, bioinformatics tools used, and their corresponding outputs are depicted.

**Fig. 2:**
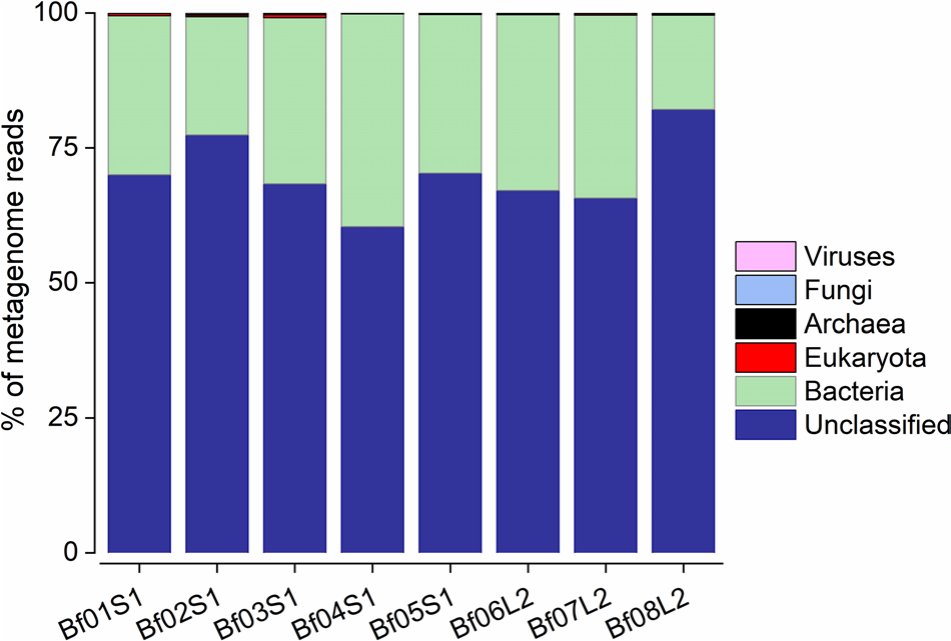
Taxonomic classification of biofloc metagenomes collected from a commercial biofloc aquaculture system. The bar plots depict the classification of metagenome reads against various NCBI RefSeq databases using Karken2 program. Percentages were calculated based on the count of reads assigned to specific taxonomic groups in relation to the total number of reads within the metagenome.

In the present study, we characterized eight metagenomes derived from the FAB community (>3 µm size fraction) of a commercial aquaculture system in South Korea that operates based on BFT. These metagenomes represent the temporal variations in the FAB community during the growth of two batches of Pacific white shrimp (*Litopenaeus vannamei*) (Table 1). A schematic diagram of the workflow followed in this study is presented in Fig. 1. The methodological workflow largely involves the collection of rearing water from a commercial biofloc aquaculture system, nucleic acid extraction from the FAB community, Illumina sequencing, and finally the bioinformatics analyses to recover MAGs. The Illumina-generated shotgun metagenome sequencing effort produced a total of 106.6 Gbp, with 12.3–16.8 Gbp per sample, and 353.18 million raw paired-end (PE) reads, with an average of 44.14 million reads per sample (Table 2). After eliminating low-quality reads and applying other quality control criteria, 300.25 million (average 37.53 million per sample) high-quality PE reads were retained. These metagenome reads exhibited a Phred quality score >30 according to the MultiQC report, indicating that the raw reads are of very good quality. The quality control criteria implemented in our study resulted in the elimination of 13.97% to 16.14% of metagenome reads across the analysed metagenomes. Taxonomic classification of the high-quality reads against various RefSeq databases revealed that a predominant fraction of metagenome reads remains unclassified. The relative proportions of these unclassified reads ranges from 60.33% to 82.10% across the biofloc metagenomes, with an average of 70.15% (Fig. 2 and Table 3). Of the classified reads, the highest proportion was attributed to bacteria (average 29.37%), followed by eukaryota (0.28%), archaea (0.10%), fungi (0.06%), and viruses (0.01%). This observation is well corroborated with a previous study that investigated the biofloc-forming community through metagenomic approach^26^.

**Table 1:** Sampling period, physicochemical properties, and inorganic nutrient content of rearing water collected from a commercial aquaculture system operating based on BFT.

Sample code	Sampling Date	Shrimp batch	Physicochemical parameters				Inorganic nutrients			
			Temp. (°C)	DO (mg/L)	Salinity (‰)	pH	Nitrite (µM)	Nitrate (µM)	TAN (µM)	Phosphate (µM)
Bf01S1	2018-04-13	Batch-1	27.52	4.10	22.44	6.90	0.18	0.40	2.30	0.55
Bf02S1	2018-04-20	Batch-1	30.16	5.20	22.62	7.08	3.28	6.00	0.14	2.29
Bf03S1	2018-04-30	Batch-1	27.43	5.55	23.15	7.06	11.75	20.00	0.20	9.00
Bf04S1	2018-05-11	Batch-1	25.75	6.98	26.91	7.92	0.70	5.60	0.70	4.60
Bf05S1	2018-05-24	Batch-1	29.03	6.23	27.41	9.05	5.58	20.00	0.70	18.00
Bf06L2	2018-06-08	Batch-2	26.78	4.60	27.17	6.37	0.34	22.20	1.10	15.40
Bf07L2	2018-06-22	Batch-2	27.65	4.09	27.30	6.18	0.17	48.40	3.60	35.40
Bf08L2	2018-07-20	Batch-2	28.16	5.27	26.47	8.20	0.98	105.00	0.50	38.00

Abbreviations: Temp., temperature; DO, dissolved oxygen; TAN, total ammonia nitrogen.

**Table 2:** An overview of the Illumina sequencing performed on the biofloc metagenomes obtained from a commercial BFT-based aquaculture system.

Sample code	Total bases (Gb)^a^	Raw PE reads (M)^b^	High-quality PE reads (M)^c^	Reads retained (%)^d^	BioSample accession number	SRA accession number
Bf01S1	12.71	42.09	36.21	86.03	SAMN34591950	SRR24442559
Bf02S1	12.81	42.44	36.27	85.47	SAMN34591951	SRR24442558
Bf03S1	16.82	55.70	47.11	84.57	SAMN34591952	SRR24442557
Bf04S1	12.30	40.74	34.23	84.01	SAMN34591953	SRR24442556
Bf05S1	12.79	42.37	36.72	84.57	SAMN34591954	SRR24442555
Bf06L2	12.51	41.45	34.76	83.86	SAMN34591955	SRR24442554
Bf07L2	13.02	43.13	36.88	85.52	SAMN34591956	SRR24442553
Bf08L2	13.66	45.26	38.07	84.13	SAMN34591957	SRR24442552

^a^Total number of nucleotide bases (Gigabases). ^b^Number of paired-end (PE) reads obtained from Illumina sequencing (million). ^c^Number of paired-end reads retained after applying quality control criteria (million). ^d^Percentage of total reads retained after applying quality control criteria.

**Table 3:** Taxonomic classification of biofloc metagenomes based on the Kraken2 program using various RefSeq databases.

Sample code	Unclassified	Bacteria	Eukaryota	Archaea	Fungi	Viruses
Bf01S1	69.99	29.48	0.43	0.05	0.03	0.02
Bf02S1	77.37	21.91	0.32	0.15	0.24	0.01
Bf03S1	68.33	30.78	0.57	0.21	0.09	0.02
Bf04S1	60.39	39.42	0.14	0.01	0.03	0.01
Bf05S1	70.30	29.41	0.18	0.07	0.03	0.01
Bf06L2	67.09	32.58	0.15	0.12	0.03	0.03
Bf07L2	65.66	33.93	0.25	0.10	0.04	0.02
Bf08L2	82.10	17.50	0.22	0.13	0.04	0.01
Average	70.15	29.37	0.28	0.10	0.06	0.01

The values represent relative abundance (%) of each taxonomic group based on the number of metagenomic reads.

Next, we used both individual assembly and co-assembly (collectively termed as “mix assembly”) approaches on our datasets (Table 4). The individual assemblies of qualified reads using SPAdes generated a total of 1,175,916 contigs with lengths of ≥1 kbp. The shortest and longest contig lengths obtained were 1.16 Mbp and 2.34 Mbp, respectively. Co-assembly produced a total of 878,328 contigs (length ≥1 kbp) with an N50 length of 3235.

**Table 4:** Overview of the assembly statistics for the analysed biofloc metagenomes.

Assembly name	No. of contigs^a^	Longest contigs^b^	N50 (bp)
Assembly-1	64097	1807760	3704
Assembly-2	123328	2130879	2694
Assembly-3	231845	1553029	2282
Assembly-4	161098	1168806	2117
Assembly-5	88492	1662365	4275
Assembly-6	185939	1334657	2339
Assembly-7	167135	2340215	2424
Assembly-8	153982	2212179	2614
Co-assembly	878328	2340215	3235

^a^Number of assembled contigs with size of ≥1000 bp. ^b^Length of the longest contig.

**Fig. 3:**
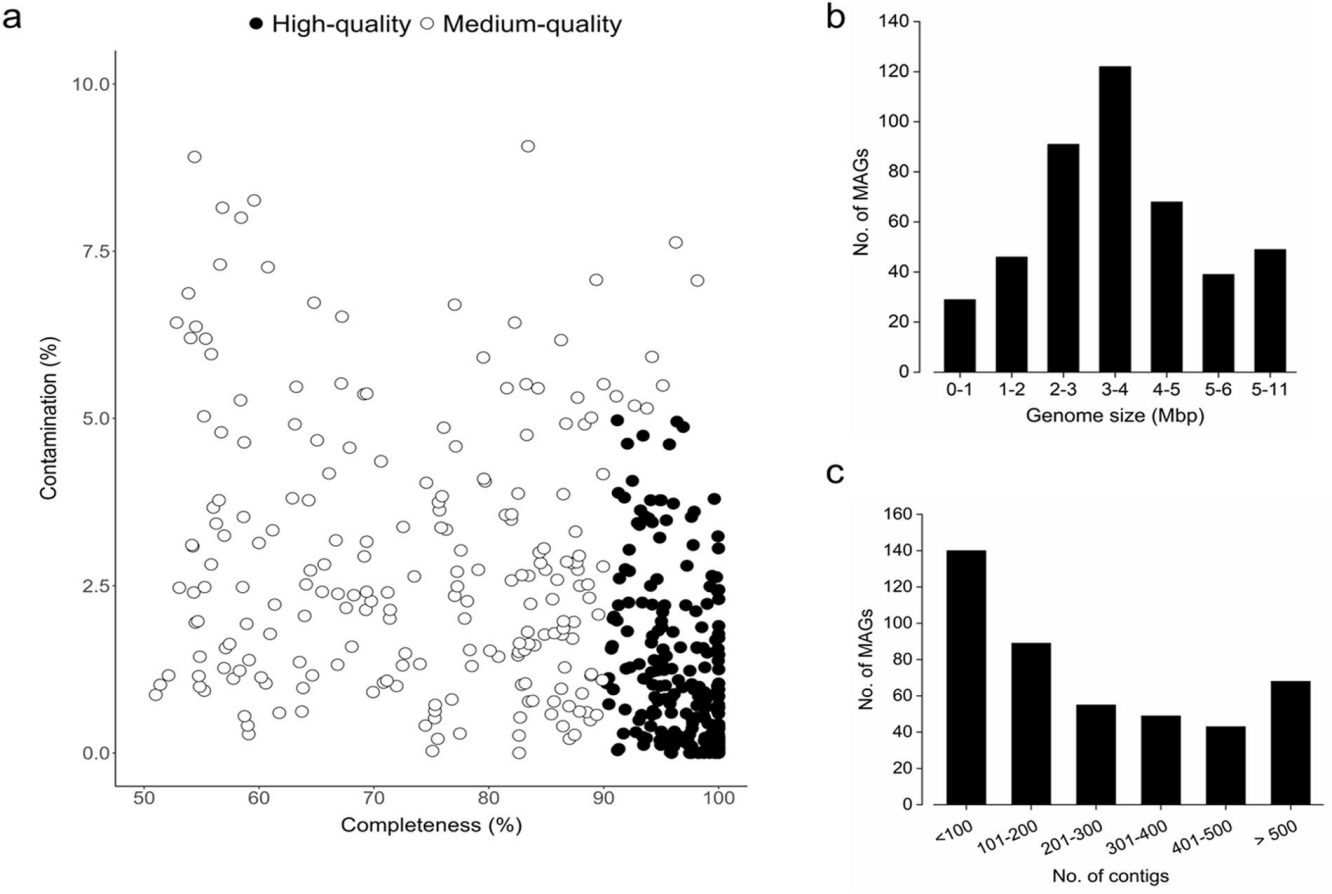
Quality metrics of MAGs recovered from the floc-associated bacterial (FAB) community. The scatter plot illustrates the distribution of the 444 recovered MAGs based on their completeness and contamination levels (**a**). Among all the MAGs, a total of 230 were classified as high-quality (≥90% completeness, ≤5% contamination), while 214 were categorized as medium-quality (≥50% completeness, ≤10% contamination). Further, the bar plots display the genome size (**b**), and the number of contigs (**c**) with respect to the number of MAGs.

We further performed binning of the contigs to recover MAGs. The bins obtained from all eight individual assemblies and one co-assembly were dereplicated at an average nucleotide identity (ANI) ≥95%, resulting in a total 444 non-redundant MAGs with completeness ≥50% and contaminations ≤10% (**see Quality Metrics File**). Among the reconstructed MAGs, 230 were classified as high-quality (completeness ≥90%; contamination ≤5%), while 214 were categorized as medium-quality (completeness ≥50%; contamination ≤10%) (Fig. 3a). All recovered MAGs had a quality score value [defined as completeness – (5 × contamination)] of ≥50. The genome sizes vary from 0.14 to 11.59 Mbp, with the majority falling within the range of 2–5 Mbp (Fig. 3b). Intriguingly, about half of the MAGs (n = 229) possessed less than 200 contigs (Fig. 3c). Of the 230 high-quality MAGs, 61 contained essential ribosomal genes, including the 16S, 23S, and 28S rRNA genes, as well as at least 18 tRNA genes (**see Quality Metrics File**). These MAGs met the stringent criteria outlined by the Genomic Standard Consortium for high-quality MAGs, ensuring their adherence to the minimum information on MAG (MIMAG) standards^27^. As expected, a higher proportion of the MAGs recovered in our study lacked ribosomal genes. This may be attributed to the inherent challenges associated with accurately assembling repetitive regions utilizing short-read sequencing methods^28^.

**Fig. 4:**
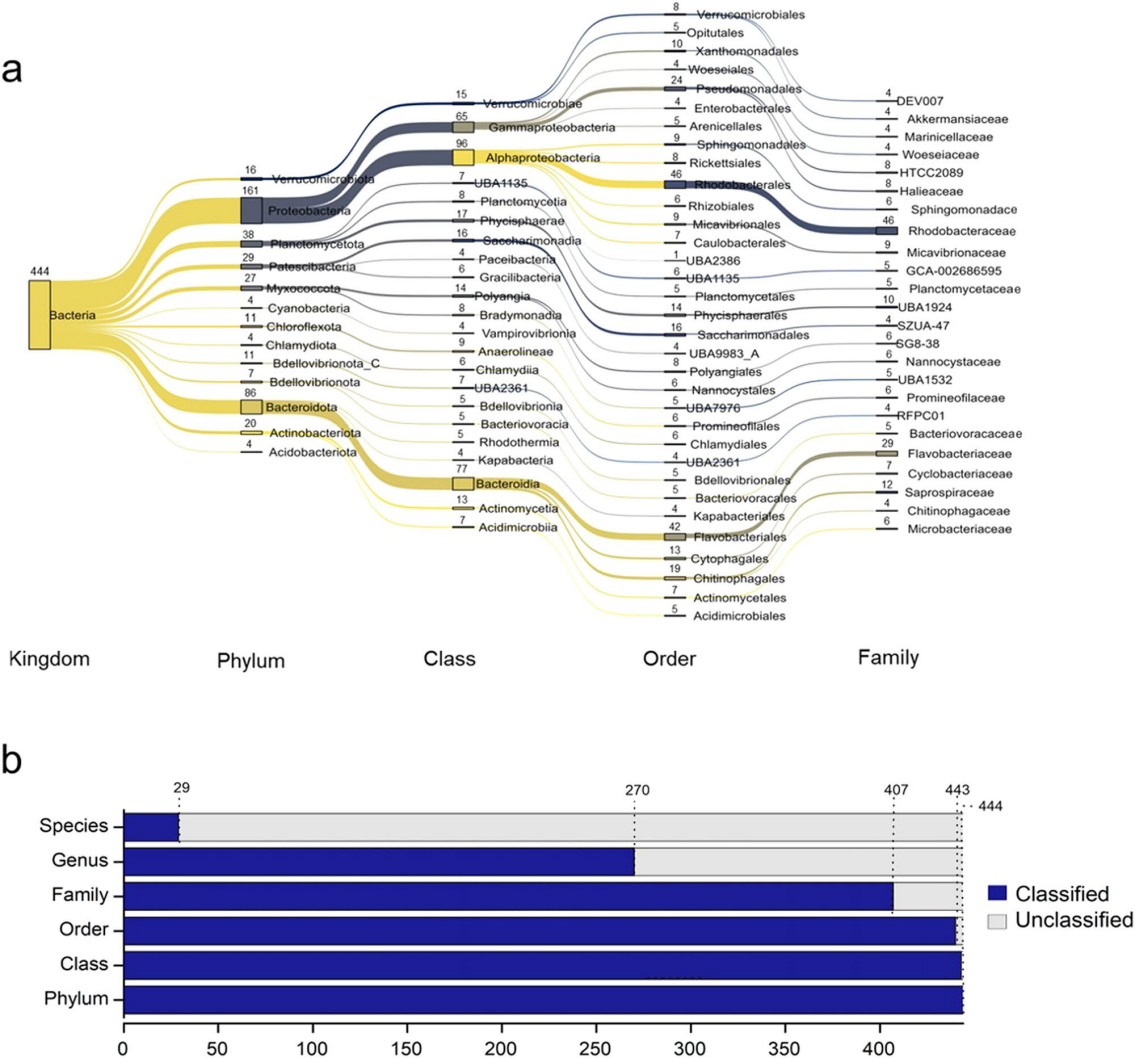
Taxonomic classification of MAGs recovered from the FAB community of a biofloc aquaculture system. The Sankey diagram provides an illustration of the classification of the dominant bacterial groups at various taxonomic ranks (**a**). Figure represents only those bacterial groups that were classified and whose abundance was represented by ≥4 MAGs. Bar plots representing the number of classified and unclassified MAGs based on GTDB at various taxonomic ranks (**b**). Detailed taxonomic classification of each MAG is provided in **Quality Metrics File**.

The taxonomic classification of the recovered MAGs revealed their distribution across nine dominant bacterial phyla, with the majority belonging to *Proteobacteria* (161 MAGs), *Bacteroidota* (86), *Planctomycetota* (38), *Myxococcota* (27), *Patescibacteria* (29), *Actinobacteriota* (20), *Bdellovibrionota* (11), *Verrucomicrobiota* (16), *Chloroflexota* (11), and *Bdellovibrionota*_C (7) (Fig. 4a**and Quality Metrics File**). Among the recovered MAGs, the family *Rhodobacteraceae* occupied a predominant proportion*, followed by Flavobacteriaceae*. The prevalence of *Rhodobacteraceae* members in biofloc aquaculture systems has been documented in earlier studies as well^29,30^. Notably, phylogenetic molecular network analysis in our recent study revealed that some *Rhodobacteraceae* members served as keystone taxa in both rearing water and bioflocs^31^. Therefore, this bacterial family may be essential component in regulating the microbial communities of various components in biofloc aquaculture systems.

Several low-abundant bacterial phyla (each represented by <10 MAGs) were also recovered from the FAB community. These phyla include *Acidobacteriota* (4 MAGs), *Chlamydiota* (6), *Armatimonadota* (2), *Calditrichota* (2), CLD3 (1), *Cyanobacteria* (4), *Delongbacteria* (1), *Dependentiae* (1), *Desulfobacterota* (2), *Eisenbacteria* (1), *Eremiobacterota* (1), *Gemmatimonadota* (3), *Hydrogenedentota* (3), and *Nitrospirota* (1) (**see Quality Metrics File**). It is intriguing to note that approximately 39% of the recovered MAGs (n = 174) could not be classified at the genus level, while 93% of the MAGs (n = 415) could not be classified at the species level (Fig. 4b). This data emphasizes the necessity of investigating aquaculture environments for microbial phylogeny.

To the best of the authors’ knowledge, this is the first report of multiple MAGs being recovered from a biofloc aquaculture system. The genome-resolved metagenomic approach employed in this study is expected to provide deeper insights into the metabolic potential and functional roles of individual microorganisms in BFT-based aquaculture systems. Gaining a comprehensive understanding of the genomic composition of biofloc-associated bacterial communities can help elucidate their roles in nutrient cycling, water quality management, disease prevention, and overall system performance. Our findings will contribute to the effective management and optimization of aquaculture systems.

## Methods

### Rearing water sampling and shotgun metagenomic sequencing

The entire methodological workflow followed in this study is represented in Fig. 1. Water samples for metagenomic analysis of the FAB community were collected from a commercial aquaculture system that uses a BFT-based approach to cultivate whiteleg shrimp (*Litopenaeus vannamei*). The investigated aquaculture system is located in Ganghwa-do, Incheon, Republic of Korea (37.7000 N, 126.3888 E). We collected surface rearing water along the growth of two *L. vannamei* batches (batch-1 and -2) on a total of eight occasions from April 2018 to July 2018 (Table 1). On each occasion, samples were collected randomly from three sites of the aquaculture tank and pooled to generate representative samples. Physicochemical characteristics such as temperature, dissolved oxygen, salinity, and pH were measured *on-site* using a handheld multi-parameter analyser YSI 556MPS (YSI Inc., Yellow Springs, USA). The concentrations of nitrite (NO_2_^−^), nitrate (NO_3_^−^), phosphate (PO_4_^3−^), and total ammonia-nitrogen (TAN, NH4^+^-N) were determined using a spectrophotometer (DR/2010, HACH Company, USA), following the standard protocol described in our previous study^32^ (Table 1). The collected samples were immediately transported to the laboratory under ice-cold conditions.

Subsequently, the water samples were centrifuged gently to separate the high-density bioflocs. The supernatant resulting from this centrifugation step was then filtered through 3 µm pore-size membrane filters (Advantec MFS, Inc., Japan) to recover any remaining low-density bioflocs^14^. Both fractions were combined and subjected to whole community nucleic acid extraction using the DNeasy PowerWater DNA isolation kit (QIAGEN, Hilden, Germany), as per the manufacturer’s instructions. The extracted metagenomic DNAs were assessed for quality and quantity using 1% agarose gel electrophoresis and a Qubit 4 Fluorometer (Thermo Fisher Scientific, USA), respectively, and preserved at −20 °C until further processing.

Illumina library preparation and the subsequent sequencing followed a standard shotgun metagenomic sequencing protocol, as detailed in a previous study^33^. In brief, DNA samples were fragmented by sonication, end-polished, A-tailed, ligated with adapter sequences. The shotgun metagenomic library was then constructed using the Nextera XT library preparation kit (Illumina, San Diego, CA, USA), in accordance with the manufacturer’s guidelines. The resulting libraries were pooled at equimolar concentrations and then sequenced on the Illumina HiSeq 2000 platform (Illumina, San Diego, CA, USA) at ChunLab, Inc. (Seoul, Republic of Korea) using a paired-end method (150 bp × 2). In total, eight metagenomes, representing FAB community at various growth stages of *L. vannamei*, were sequenced from a biofloc aquaculture system.

### Quality enhancement, taxonomic classification, and assembly of metagenomes

Forward and reverse Illumina raw reads were initially visualized using MultiQC v1.11^34^, followed by processing through BBduk program from the BBTools suits v39.01^35^. Adapters were trimmed, contaminants were screened, and short-length reads were removed using the following parameters: k=23, ktrim=r, mink=11, hdist=1, tpe, tbo, ftm=5, qtrim=rl, trimq=20, and minlen=100. The resulting high-quality reads were initially subjected to taxonomic classification against various preconstructed databases (https://benlangmead.github.io/aws-indexes/k2), including RefSeq archaea, bacteria, viruses, plasmids, human, UniVec Core, protozoa, and fungi, using Kraken2 program v2.1.3^36^.

On the other hand, obtained high-quality reads were assembled into longer fragments using metaSPAdes v3.15.4 with k-mer values of 21, 33, 55, 77, 99, and 127^37^. Both individual assembly and co-assembly approaches (collectively referred as the “mix-assembly” approach)^38^ were applied to our dataset. The individual assembly was used to obtain high-quality genomes from fairly-abundant bacterial groups, while the co-assembly approach was employed to recover genomes from less abundant bacteria^39,40^. The adapted assembly approaches provided eight individual assemblies and one co-assembly. Finally, we utilized metaQUAST v5.1.0^41^ to evaluate quality metrics and statistics of each metagenome assembly.

### Reconstruction of MAGs and taxonomic assignment

Contigs with a length >1 kb were binned to recover MAGs using the metaWRAP v1.3.2 pipeline^42^. During the metaWRAP processing, the binning module was deployed to generate the initial bin sets based on reads coverage and tetranucleotide frequencies. Subsequently, the bin_refinement module (parameters: -c 50, -x 10) was employed to recover consolidated sets of bins. The multiple bin sets recovered from all eight individual assemblies and one co-assembly were de-replicated using dRep v3.4.2 with a 95% ANI threshold to remove redundant bins and retain only the highest quality ones^39^. Default parameters were used for dRep, except for -comp 50. The final non-redundant collection of MAGs, showing medium- to high-quality (completeness ≥50%; contamination ≤10%), was retrieved after a quality evaluation using CheckM2 v1.0.1^43^, according to the proposed definition of MIMAG^27^. CheckM2, the program employed here, is renowned for estimating the completeness and contamination of microbial genomes, courtesy of a set of lineage-specific marker genes. Additional quality control measures were enforced to ensure the recruitment of only high-quality MAGS. Specifically, we selected MAGs with a quality score ≥50, calculated by deducting five times contamination from the completeness^44^. In addition, ribosomal RNA genes and transfer RNA genes were detected using Barrnap v0.9 (https://github.com/tseemann/barrnap) and tRNAscan-SE v2.0.9^45^, respectively.

Of a high number of initially reconstructed bins (approximately 950), a total of 444 passed the imposed quality control criteria and therefore were considered as MAGs (**see Quality Metrics File**). These MAGs were named using the following scheme: the characters preceding the term ‘bin’ represent the assembly from which they were binned (‘1’ to ‘8’ for individual assemblies and ‘Co’ for co-assembly), and the numerical value following the term ‘bin’ corresponds to the number of non-redundant MAGs within each assembly. A comprehensive overview of various statistics, including completeness, contamination, genome size, GC content, positions of the ribosomal RNA genes, and the number of contigs of the recovered 444 MAGs, is detailed in **Quality Metrics File** and summarized in Fig. 3. Finally, the MAGs were taxonomically assigned against the Genome Taxonomy Database (GTDB; release R207_v2) using the Genome Taxonomy Database toolkit (GTDB-Tk) v2.2.4 (options: --full_tree, --skip_ani_screen)^46^. The entire bioinformatics roadmap used for the reconstruction and taxonomic classification of MAGs is illustrated in Fig. 1.

## Data Records

The shotgun metagenome reads generated in this study are publicly available on the NCBI Sequence Reads Archive (SRA) under BioProject identifier PRJNA967453^47^ and accession number SRP436034^48^. The reconstructed MAGs have been deposited in the DDBJ/ENA/GenBank database under accession numbers JAUHVK000000000–JAUIML000000000, and their fasta files have been made accessible through figshare^49^. Detailed information pertaining to all the reconstructed MAGs, including their corresponding BioSample and GenBank accession numbers, is detailed in **Quality Metrics File**^49^.

## Technical Validation

The removal of contaminant bases, adapter sequences, and short-length reads was performed using BBduk. The final read sets were then visualized using MultiQC. We selected only those reads that had a quality score ≥30, suggesting that the majority of analysed metagenome reads were of high-quality. In adherence to the MIMAG guidelines, the quality of recovered MAGs was assessed using CheckM2 for their completeness and contamination. We only selected those MAGs that met the specified quality thresholds (as presented in **Quality Metrics File**). As an additional measure of quality, we identified the presence of tRNA and rRNA genes in all MAGs using tRNAscan-SE and Barrnap, respectively.

## Data Availability

All software used, with versions and non-default parameters, is described precisely and referenced in the method section to ensure easy access and reproducibility. For further transparency, the complete set of codes employed throughout the bioinformatics workflow have been uploaded to a GitHub repository at https://github.com/Meora-Rajeev/Biofloc-Metagenomics^50^.
